# CBCR: A Curriculum Based Strategy For Chromosome Reconstruction

**DOI:** 10.3390/ijms22084140

**Published:** 2021-04-16

**Authors:** Van Hovenga, Oluwatosin Oluwadare

**Affiliations:** 1 Department of Mathematics, University of Colorado Colorado Springs, Colorado Springs, CO 80918, USA; vhovenga@uccs.edu; 2 Department of Computer Science, University of Colorado Colorado Springs, Colorado Springs, CO 80918, USA

**Keywords:** genome structure, 3D chromosome structure, Hi-C, curriculum learning, optimization

## Abstract

In this paper, we introduce a novel algorithm that aims to estimate chromosomes’ structure from their Hi-C contact data, called Curriculum Based Chromosome Reconstruction (CBCR). Specifically, our method performs this three dimensional reconstruction using *cis-chromosomal* interactions from Hi-C data. CBCR takes intra-chromosomal Hi-C interaction frequencies as an input and outputs a set of xyz coordinates that estimate the chromosome’s three dimensional structure in the form of a .pdb file. The algorithm relies on progressively training a distance-restraint-based algorithm with a strategy we refer to as curriculum learning. Curriculum learning divides the Hi-C data into classes based on contact frequency and progressively re-trains the distance-restraint algorithm based on the assumed importance of each curriculum in predicting the underlying chromosome structure. The distance-restraint algorithm relies on a modification of a Gaussian maximum likelihood function that scales probabilities based on the importance of features. We evaluate the performance of CBCR on both simulated and actual Hi-C data and perform validation on FISH, HiChIP, and ChIA-PET data as well. We also compare the performance of CBCR to several current methods. Our analysis shows that the use of curricula affects the rate of convergence of the optimization while decreasing the computational cost of our distance-restraint algorithm. Also, CBCR is more robust to increases in data resolution and therefore yields superior reconstruction accuracy of higher resolution data than all other methods in our comparison.

## 1. Introduction

The nucleus of each eukaryotic cell stores important information about an organism’s genetic makeup in the form of chromosomes [1]. Each chromosome is made of DNA, and the set of all chromosomes is known as the genome. There are well known connections between the makeup of an organism’s genome and the traits it possesses, for example. hair color, skin color, propensity for diseases, and so forth [1]. One particularly affective attribute of a cell’s genome is the shape and spatial occupation of its respective chromosomes. The organization of chromosomes facilitates inter-gene communication and regulation, thus contributing to the stability of a genome [2]. Thus, research into the capturing of topological makeup of chromosomes is valuable.

For this reason, chromosome conformation capturing techniques such as 3C [3], 4C [4], 5C [5], and Hi-C [6,7,8] were developed to analyze the spatial organization of chromatin in a cell. Hi-C is a method that utilizes biotin-labeled nucleotides in the pre-ligation step of 3C to ensure that only ligation junctions are analyzed [6]. The fragments are then analyzed using massively parallel DNA sequencing to generate reads [6]. These reads are compared to the original genome, and each fragment is given a score based on the quantity of matching nucleotides. This results in a contact matrix that describes the interaction frequencies of the fragments with the original genome. The main advantage to Hi-C analysis is the fact that each locus of the genome is compared to each other locus which allows for insight into its global structure.

Due to the high quantity of data that is produced with the Hi-C method, Hi-C data is typically used to make inferences of the three-dimensional (3D) structure of chromosomes. Several methods have been developed for the reconstruction of the chromosomal topology from contact data [9]. One major class of these methods is called the distance-restraint method [9,10]. Distance-restraint methods aim to optimize an objective function with respect to restraints derived from the contact data to find a chromosomal structure that best suits the contact data [11,12,13]. Usually, distance-restraint methods use a function to convert the contact matrix to a distance matrix, known as wish distances, and use these distances as the constraints for the optimization [9]. This is typically done by randomly initializing xyz coordinates that correspond to each locus in the chromosome and training these coordinates to best recreate the wish distances of the input from their respective pairwise Euclidean distances. In this paper, we introduce a distance-restraint method for 3D genome reconstruction from intra-chromosomal Hi-C contact data that is inspired by a curriculum-based strategy. The method will be referred to as Curriculum Based Chromosome Reconstruction (CBCR).

Curriculum learning is a method of training machine learning models that is inspired by the way in which humans learn [14]. Human education is structured based on a progressive construction of topics ordered by increasing difficulty. For example, when children learn basic math, the concept of counting is introduced first. From counting, one can learn addition and subtraction. From addition, one can learn multiplication, and from multiplication, division can be learned. This concept is known as a curriculum. The same strategy has been implemented in machine learning as well. The general idea behind curriculum learning in the context of machine learning is to divide a global task into several smaller subtasks that vary in terms of difficulty but are connected based on transferable knowledge. From this division, one can train a model beginning with the easiest sub-tasks and work progressively on more difficult tasks until the global task can be accomplished. This concept of curriculum learning is the core aspect of CBCR.

In this paper, we rigorously describe the construction of CBCR. We then test CBCR on both simulated and real Hi-C data. We further validate the performance of CBCR by utilizing comparisons with FISH, HiChIP, and ChIA-PET data. We show that the addition of curricula to our distance-restraint algorithm increases its time performance and accelerates the rate of convergence of the optimization. Our tests show that CBCR is also more robust to increases in the resolution of the input data when compared with other standard algorithms. We hope that this paper inspires the use of curricula learning in other distance-restraint algorithms as an effective method to accelerate convergence, reduce computational load, and increase performance on higher resolution input data.

## 2. Materials and Methods

CBCR relies on a distance-restraint algorithm to convert Hi-C interaction frequency data to into a 3D structure. For the distance restraint algorithm we use a scaled maximum likelihood as an objective function and utilize gradient ascent to optimize. For ease of notation, we refer to the distance-restraint algorithm as DR(S,D), where *S* is an initial structure composed of xyz coordinates and *D* is a set of input distances with which we train the structure *S*.The outputs of DR are represented as $DR\left(S,D\right)=\left(S^{\prime },D^{\prime }\right)$ where $S^{\prime }$ and $D^{\prime }$ are the output structure and distances respectively derived from training on *D*. The general method will be as follows. Assume we have a Hi-C data set, D, of *N* contact regions. Firstly, we convert the Hi-C contact data D into a set of distances *D*. We then divide these corresponding distances *D* into *n* smaller subsets, $C_{0},C_{1},C_{2},\cdots ,C_{n}$ based off of information that is assumed to be most pertinent to the 3D structure of the genome; these assumptions are described in Section 2.1. We refer to these subsets as the *curricula*. The curricula are ranked from least difficult to most difficult. We then train a randomly initialized structure, $S_{0}$ using the first, easiest curriculum, $C_{0}$, as an input to the distance-restraint algorithm to generate a new structure, $S_{1}$, and new distances, $C_{0}^{\prime }$ corresponding to the pairwise Euclidean distances from $S_{1}$, from $DR(S_{0},C_{0})$. We then concatenate $C_{0}^{\prime }$ with $C_{1}$ to generate the next set of training data $C_{0,1}$. Following this, we train $S_{1}$ using the data from $C_{0,1}$ to generate $DR\left(S_{1},C_{0,1}\right)=\left(S_{2},C_{0,1}^{\prime }\right)$. This process will continue until each curriculum has been input into DR.

**Figure 1:**
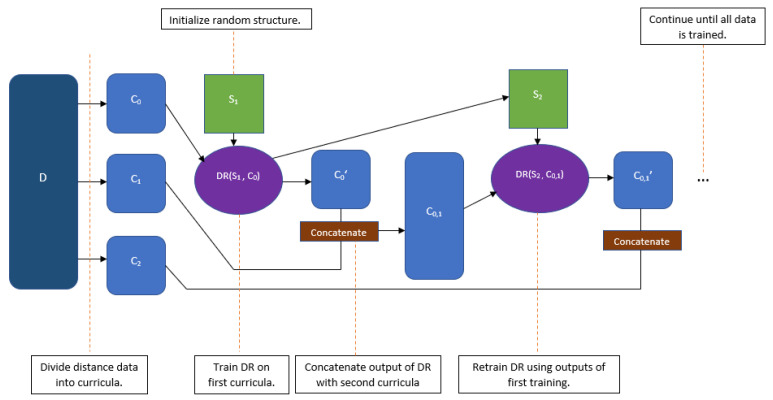
Flow diagram for Curriculum Based Chromosome Reconstruction (CBCR).

Essentially, we re-train structures generated by the distance-restraint algorithm multiple times in order to generate an increasingly refined predicted 3D chromosomal structure. This process is depicted visually in Figure 1.

### 2.1. Division of Curricula

This method begs the question of which Hi-C data is most pertinent to the 3D structure of the chromosome; i.e., what is easy data and what is hard data? Most curriculum-based machine learning algorithms rely on accuracy metrics to rank the difficulty of certain subsets; data that generates low predictive accuracy is ranked as more difficult. This is impossible for the task of genome reconstruction, however, because there are no labels or categories that can allow for measurements of predictive accuracy. For this reason, the guiding assumption of this algorithm is that data with a higher contact frequency is more pertinent to the 3D structure of the genome. From this assumption, we can divide the Hi-C data into subsets of ranging difficulty based on contact frequency. To divide the curricula, we order the data based on increasing contact frequency and divide the resulting set into equal bins so that the first bin has the highest contact frequencies and the last has the lowest. The higher contact bins are considered easier and are therefore utilized as earlier training data. See Section 3.1 for details on finding the optimal number of curricula for this division.

### 2.2. Conversion of Interaction Frequencies to Distances

As CBCR is a distance-restraint method, we must convert the interaction frequencies of the Hi-C data to hypothetical Euclidian distances, also known as wish distances. The relationship between distance and interaction frequencies has been shown to be inversely exponential both empirically [8,15,16,17], and theoretically [18,19]. In this work, we assume the typical inverse relationship utilized in [20]; i.e., we convert based on the formula

(1) $$d\left(i,j\right)=\frac{1}{IF_{i,j}^{\gamma }},$$

where $IF_{ij}$, is the interaction frequency between sites *i* and *j*. Here, γ is a hyperparameter called the conversion factor. In general, the value for γ is unknown and varies depending on the underlying chromosome. It has been shown, however, that γ lies in the range [0.1,2] for most common cell types [19]. Thus, in this work, we assume that the optimal conversion belongs to the interval [0.1,2] and perform a grid search in this interval to find the optimal model.

### 2.3. Objective Function

In addition to the division of the contact data into curricula, CBCR will also modify the objective function proposed by Oluwadare et al., 2018, 3DMax [11]. The objective function used in the 3DMax algorithm is the log likelihood of the distance data D given some unknown chromosomal structure S where the distribution of D is assumed to be normal. We modified this likelihood in such a way that more probabilistic mass is assigned to easy curricula and less is assigned to difficult curricula. The logic for this assumption is that the easy curricula, i.e., curricula with higher contact values, is assumed to be more pertinent to the 3D reconstruction of the chromosome and therefore should have more influence on the likelihood function. In order to accomplish this, we scale the assumed probabilities of our contact data based on a factor that depends on the proportion of data that has previously been utilized for training in the current curriculum. In this work, we refer to data that has been previously used as training data for the distance-restraint algorithm as previously *seen* data.

Assume that we divide our data into n curricula $\left\{C_{1},C_{2},\cdots ,C_{n}\right\}$. Let $S_{k}$ be the structure at curriculum *k* and denote the distance corresponding to $S_{k}$ by $Dt^{\prime }\left(k\right)=\left\{dt_{1}^{\prime }\left(k\right),\cdots ,dt_{r}^{\prime }\left(k\right)\right\}$. Since these distances were generated from a trained structure, we refer to them as *trained distances*. Similarly, denote the untrained distance data in curriculum *k* by $D^{\prime }\left(k\right)=\left\{d_{1}^{\prime }\left(k\right),\cdots ,d_{s}^{\prime }\left(k\right)\right\}$. Since the distances in $D^{\prime }\left(k\right)$ have not been optimized by DR, they correspond to the distances induced by the first, randomly initialized structure, $S_{0}$. Note that $r=\left|C_{1}\right|+\cdots +\left|C_{k-1}\right|$ and $s=\left|C_{k}\right|$. Let $p_{k}=\frac{r}{r+s}$ and $q_{k}=\frac{s}{r+s}$ be the proportion of trained and untrained data respectively at curriculum k. Finally, let $Dt\left(k\right)=\left\{dt_{1}\left(k\right),\cdots ,dt_{r}\left(k\right)\right\}$ and $D\left(k\right)=\left\{d_{1}\left(k\right),\cdots ,d_{s}\left(k\right)\right\}$ be the wish distances corresponding to the trained and untrained data respectively. Then we define the scaled likelihood as

(2) $$L\left(S\right)=\prod_{i=1}^{r}{P(Dt(k)|S)}^{\propto p_{k}}\cdot \prod_{j=1}^{s}{P(D(k)|S)}^{\beta q_{k}},$$

where *P* is the Gaussian distribution conditional on structure *S*. Here, *S* represents the hypothetical true structure of the chromosome. The parameters α∈[0,1] and β=1−α are trainable, scaling hyperparameters (see Section 2.4). We assume that Dt and *D* are conditionally independent of *S* in order to derive (2). Since *P* is assumed to be Gaussian, L(S) takes the form

(3) $$L\left(S\right)=\;{\frac{1}{\sigma \sqrt{2\pi }}}^{\alpha r+\beta s}exp\left[-\frac{1}{2\sigma^{2}}\left(\alpha p_{k}\sum_{i=1}^{r}{\left({dt\prime }_{i}\left(k\right)-{dt}_{i}\left(k\right)\right)}^{2}+{\beta q}_{k}\sum_{i=1}^{s}{\left({d\prime }_{i}\left(k\right)-d_{i}\left(k\right)\right)}^{2}\right)\right].$$

Taking the log of *L* and letting

(4) $$\sigma =\sqrt{\frac{\alpha p_{k}\sum_{i=1}^{r}{\left({dt\prime }_{i}\left(k\right)-{dt}_{i}\left(k\right)\right)}^{2}+{\beta q}_{k}\sum_{i=1}^{s}{\left({d\prime }_{i}\left(k\right)-d_{i}\left(k\right)\right)}^{2}}{r+s}},$$

we get

(5) $$\log\left(L\left(S\right)\right)=-\left(\alpha r+\beta s\right)\;\log\left(\sqrt{\frac{\alpha p_{k}\sum_{i=1}^{r}{\left({dt\prime }_{i}\left(k\right)-{dt}_{i}\left(k\right)\right)}^{2}+{\beta q}_{k}\sum_{i=1}^{s}{\left({d\prime }_{i}\left(k\right)-d_{i}\left(k\right)\right)}^{2}}{r+s}}\right)-\frac{r+s}{2}.$$

This is the final likelihood function that we aim to optimize.

### 2.4. Optimization

In order to estimate the 3D structure, we maximize the log likelihood function (5). Our maximization relies on gradient ascent. Specifically, we utilize adaptive gradient ascent, or AdaGrad [21], to optimize the xyz coordinates corresponding to $Dt^{\prime }\left(k\right)$ and $D^{\prime }\left(k\right)$ and adaptive moment estimation, or Adam [22], to optimize α and β.

AdaGrad is an iterative optimization technique that adapts the learning rate for each parameter according to sum of previous gradients [21]. This per-parameter update scheme creates larger updates for sparse parameters and smaller updates for less sparse parameters. The result is a learning rate that decreases as training progresses depending on the contribution of each parameter to the loss function. This often results in improved convergence for sparse data. The mathematical formulation of Adam is as follows. Let λ be an initial learning rate and $S_{i}^{t}$ be the estimated structure from the set of distances *D* at time *t*. At each iteration *t*, we compute the gradient of $\log(L\left(S_{i}^{t}\right))$ with respect to each *x*, *y*, and *z* coordinate in *D* (see Appendix A.1 for the full computation). That is, we compute

$$\nabla \log\left(L\left(S_{i}^{t}\right)\right)=g_{i,t},$$

and we update $S_{i}^{t}$ according to

$$S_{i}^{t+1}=S_{i}^{t}+\frac{\lambda }{\sqrt{G_{t,i}+\epsilon }}\cdot g_{i,t},$$

where $G_{t,i}$ is the sum of the squared gradients of component *i* up to time *t* and λ is the learning rate. That is,

$$G_{i,t}=\sum_{k=1}^{t}{\left(g_{i,k}\right)}^{2}.$$

Here, ϵ is a small term meant to prevent division by zero. In our experiments, we used ϵ=0.0001. This choice was guided by the findings in [11].

Like AdaGrad, Adam is an adaptive, iterative optimization technique. Adam, however, relies on the estimated first and second moments of component-wise gradients to adapt the learning rate to the data [22]. This second moment estimator prevents the learning rate λ from decreasing too quickly. We utilized Adam to optimize the parameter α during training. In order to do this, we first compute the gradient of the log likelihood with respect to α (see Appendix A.2 for the full computation). That is, we compute

$$\frac{\partial }{\partial \alpha }\log\left(L\left(S_{i}^{t}\right)\right)=g_{i,t}.$$

We then update the biased first, $m_{i,t+1}$, and second, $v_{i,t+1}$ moment estimators using

$$\begin{array}{cc} m_{i,t+1} & =\gamma_{1}m_{i,t}+\left(1-\gamma_{1}\right)g_{i,t}\\ v_{i,t+1} & =\gamma_{2}v_{i,t}+\left(1-\gamma_{2}\right)g_{i,t}^{2}, \end{array}$$

where we initialize the first and second moments at time t=1 using $m_{i,1}=v_{i,1}=0$. Here, $\gamma_{1},\gamma_{2}\in \left[0,1\right]$ are hyperparameters that change the bias of the first and second moment estimates. Following this computation, we find the bias-adjusted first and second moment estimates using

$$\begin{array}{cc} {\hat{m}}_{i,t+1} & =\frac{m_{i,t}}{\left(1-\gamma_{1}^{t}\right)}\\ {\hat{v}}_{i,t+1} & =\frac{v_{i,t}}{\left(1-\gamma_{2}^{t}\right)} \end{array}$$

Finally, we update $\alpha_{t}$ according to

$$\alpha_{t+1}=\alpha_{t}+\lambda \frac{{\hat{m}}_{i,t+1}}{\sqrt{{\hat{v}}_{i,t+1}+\epsilon }}.$$

In this work, we use $\gamma_{1}=0.9$ and $\gamma_{2}=0.999$ as recommended in [22]. Moreover, we chose a learning rate of 0.2 for all trials. This choice was guided by the findings in [11].

The advantages to using Adagrad and Adam as optimizers is that the distance-restraint algorithm is less dependent on the initial choice of learning rate. Since each optimizer decreases the learning rate according to the changes in gradients over time, we are less likely to run into convergence issues due to a high learning rate. One drawback of the Adagrad optimizer, however, is that the learning rate can decrease too quickly, thereby leading to sub-optimal convergence; a problem that is less likely to occur with Adam [22]. For this reason, we initially attempted to optimize both the distance data and α using Adam as an optimizer. Unfortunately, this led to unstable convergence. Thus, we chose to use Adagrad for the distance optimizer and Adam for the α optimizer.

### 2.5. Early Stopping Criteria

To bound the run time for CBCR, we utilize early stopping criteria, the first of which is a maximum number of iterations. This is a value set by the user that determines how many parameter updates will take place before CBCR is stopped. We will refer to this value by $max\_iter_{0}$. This value also determines the number of iterations per curriculum. See Appendix B for a detailed procedure of how we find the number of iterations per curriculum from $max\_iter_{0}$. The second stopping criterion is a convergence threshold between the sub-curricula. This threshold is measured by the absolute difference of the dSCC (see Section 2.7) between two consecutive sub-curricula. If the absolute difference is less than the threshold for two consecutive curricula, then all of the remaining data is lumped together with the previously seen data and optimized for a final maximum number of iterations. Formally, let $dSCC_{i}$ be the dSCC value for sub-curriculum *i* and $convg_{0}$ be a user-set convergence threshold. Then if $|dSCC_{i-1}-dSCC_{i}|<convg_{0}$, we set $C_{i+1}=\cup_{j=i+1}^{n}C_{j}$ and we proceed to iterate over D(i+1) and Dt(i+1) accordingly for a final number of iterations determined by the user. We will hereon refer to this final number of iterations by $max\_iter_{1}$.

**Figure 2:**
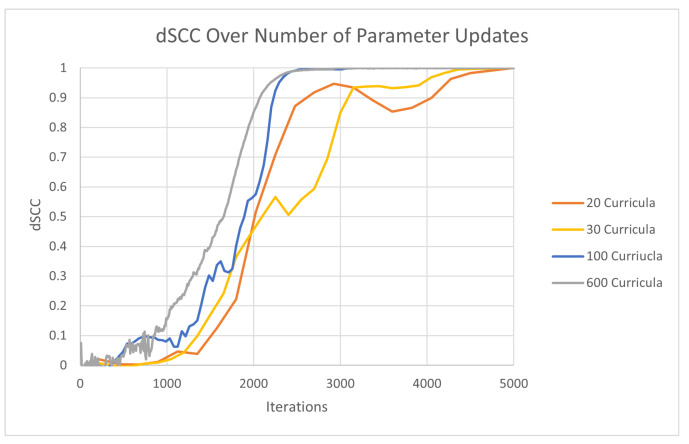
Evolution of distance Spearman Correlation Coefficient (dSCC) over number of iterations for 20, 30, 100, and 600 curricula.

It is unnecessary to calculate the dSCC for every sub-curriculum for two reasons. Firstly, the first few curricula do not contain enough data for the generated structure to converge (see Figure 2). Secondly, for larger values for the total number of curricula, there is not enough data added to each curriculum to change the dSCC significantly, so the absolute difference between the dSCC of two consecutive sub-curricula may be below the convergence threshold even though the generated structure has not converged. To resolve these issues, we only evaluate sub-curricula dSCC after half of the data has been previously seen. Moreover, we only allow CBCR to evaluate sub-curricula dSCC for a maximum of 20 times and allocate the frequency of this evaluation with respect to the number of curricula accordingly.

Finally, we utilize an intra-curriculum convergence threshold based upon the changes in gradient for a given curriculum. Specifically, we would like to stop iteration in a given curriculum if the gradient and loss are no longer changing significantly. To do this, we evaluate the $l_{2}$ norm of the gradients at each time iteration and compare it with a proportion of the loss. That is, we calculate $\left|\right|G_{t}{\left|\right|}_{l_{2}}=\sqrt{\sum_{i=1}^{r+s}g_{i,t}^{2}}$ and if $\left|\right|G_{t}{\left|\right|}_{l_{2}}<convg_{1}\cdot loss\left(t\right)$, we know that that the gradients are no longer changing significantly, so we stop iterating over the current curriculum and move to the next. Here, $convg_{1}$ is another convergence threshold and loss(t) is the loss at iteration *t*.

### 2.6. Data

#### 2.6.1. Simulated Hi-C Data

We evaluated the performance of CBCR on simulated Hi-C contact maps generated by Zou et al. [23]. The contact maps are based on a regular helical structure. Specifically, we assessed the performance on the helical structures at 90% signal coverage. Here, the signal coverage refers to the percentage of non-zero entries in the contact map. Also, we evaluated the performance of CBCR on a simulated yeast chromosome developed by Duan et al. [24]. The simulated yeast chromosome is represented by 610 contact points at 50 kb resolution and no noise. The advantage to evaluating on a simulated dataset is that the underlying structure that we wish to predict is known. This allows for direct comparison between the predicted structure and the actual structure which is not possible when testing on Hi-C data generated from actual chromosomes since their underlying structure is usually unknown.

#### 2.6.2. Real Hi-C Data

In addition to evaluation on the synthetic data sets, we also analyzed the performance of CBCR on real Hi-C data. The benefit of evaluating the performance on real Hi-C data is that the real data allows us to test how the algorithm responds to features that are inherent in actual Hi-C data, such as excessive noise, biases, and complicated structures, that may not be present in the synthetic data. Also, by comparing the algorithm at different resolutions, we can see how performance is affected by the amount of available data. We used the GM12878 cell line from [25] downloaded from the Genome Structure Database (GSDB) repository [26] under the GSDB ID OO7429SF. To reduce the effects of noise in the Hi-C data, each cell line was normalized using the Knight-Ruiz normalization technique [27]. There are three Hi-C maps from [25] for the GM12878 line: primary, replicate, and combined. The primary and replicate maps were acquired from two separate experiments, and the combined (primary + replicate) map was generated from the combination of the two experiments. We first tested the performance of CBCR on all 23 chromosomes at four coverage levels utilizing the combined mappings: 1 mb, 500 kb, 250 kb, and 100 kb. Unlike the simulated data, there is no known structure associated with the real Hi-C data. For this reason, the generated structures were compared to the wish distances generated by the corresponding interaction frequency contact matrix. We also generated structures using CBCR with the primary and replicate maps separately for each chromosome at 1 mb resolution. We then compared the outputs from the primary mappings to the outputs from the secondary mappings in order to test for reproducibility of the biological replicates.

For our main experiments, we used data acquired using the Mbol restriction enzyme. The Mbol is a 4-cutter restriction enzyme, cutting a 4 bp long sequence: GATC. Hence, to evaluate the CBCR’s reproducibility, we also compared the output generated for Hi-C data for the same cell type but with different restriction enzymes. We compared the generated structures from contact matrices generated from Mbol based Hi-C protocol with output structures from contact matrices generated from Dpn-II restriction enzyme, a 4-bp cutter–based Hi-C protocol [25].

#### 2.6.3. ChIA-PET and HiChIP Data

Chromatin immunoprecipitation (ChIP) is a technique to investigate protein specific interactions in chromosomes. ChIP relies on antibodies to precipitate specific proteins, histones, or transcription factors from cell populations [28]. ChIP can also be combined with sequencing technologies to quantify these interactions. Chromosome Interaction Analysis by Paired-End Sequencing (ChIA-PET) [29] and HiChIP [30] are two ChIP sequencing techniques. ChIA-PET and HiChIP both utilize antibodies to enrich chromatin that are bounded by a specific protein. The main difference between HiChIP and ChIA-PET is that the antibodies in HiChIP are introduced following an in situ preparation of a Hi-C contact library in order to reduce the chance of false-positive interactions generated by the ChIP step. Following the enrichment steps of HiChIP and ChIA-PET, chromosomes are cut using a restriction enzyme, re-ligated, and sequenced just as in Hi-C experiments. The chromatin enrichment step ensures that only regions of the genome that possess the binding-site associated with the protein of interest may be cross-linked so that only protein-specific interactions are measured. Thus, ChIA-PET and HiChIP can identify loops in the 3D structure of a chromosome that may be especially pertinent to genome function by choosing to precipitate proteins that are known to be important in gene regulation.

For additional validation of our results, we used the H3K27ac loop calls from GM12878 cell HiChip dataset from Mumbach et al., 2017 [31] also used in study by Bhattacharyya et al. [32]. The H3K27ac is a histone post-translational modification enriched in cis-regulatory regions, in particular promoters, and is regarded as an active enhancer mark [33]. We also utilized ChIA-PET data from the NCBI GEO database (GEO accession: GSE72816) for the RNAPII ChIA-PET data from human GM12878 cells [34].

#### 2.6.4. FISH Data

Fluorescent in situ hybridization (FISH) is a technique for directly observing chromosome structure. In FISH, specific DNA fragments are colored using a fluorescent dye and are then attached to a chromosome using in situ hybridization [35,36]. The chromosome is then observed microscopically. The fluorescent dye allows for the specific DNA fragments to be identified and analyzed. To further validate our results, we used the FISH data provided by Rao et al. [25]. This particular FISH data measures the distance between three peaks called from their Hi-C maps of chromosomes 11, 13, 14, and 17 of the GM12878 cell line.

### 2.7. Evaluation

In order to evaluate the performance of CBCR, we used two metrics: time performance and reconstruction accuracy. Reconstruction accuracy refers to how accurately the pairwise distances between each chromosome locus corresponding to the structure generated by CBCR match the actual pairwise distance of the underlying chromosome from which the input Hi-C data was generated. To measure this similarity, we use the distance Spearman Correlation Coefficient (dSCC), the distance Pearson Correlation Coefficient (dPCC), and the distance Root Mean Square Error (dRMSE). These metrics were shown by [13] to be valid benchmarks for the accuracy of distance-restraint based chromosome reconstruction methods. These three formulae are given by

(6) $$dPCC=\frac{\sum_{d_{i}^{\prime }\in D^{\prime }}\left(d_{i}^{\prime }-\bar{d^{\prime }}\right)\sum_{d_{i}\in D}\left(d_{i}-\bar{d}\right)}{\sqrt{\sum_{d_{i}^{\prime }\in D^{\prime }}{\left(d_{i}^{\prime }-\bar{d^{\prime }}\right)}^{2}\sum_{d_{i}\in D}{\left(d_{i}-\bar{d}\right)}^{2}}}$$

(7) $$dSCC=\frac{\sum_{d_{i}^{\prime }\in D^{\prime }}\left(rank\left(d_{i}^{\prime }\right)-\bar{rank(d^{\prime })}\right)\sum_{d_{i}\in D}\left(rank\left(d_{i}\right)-\bar{rank(d)}\right)}{\sqrt{\sum_{d_{i}^{\prime }\in D^{\prime }}{\left(rank\left(d_{i}^{\prime }\right)-\bar{rank(d^{\prime })}\right)}^{2}\sum_{d_{i}\in D}{\left(rank\left(d_{i}\right)-\bar{rank(d)}\right)}^{2}}}$$

(8) $$dRMSE=\sqrt{\frac{1}{\left|D\right|}\sum_{d_{i}\in D,d_{i}^{\prime }\in D^{\prime }}{\left(d_{i}^{\prime }-d_{i}\right)}^{2}},$$

where $D^{\prime }$ is the set of pairwise distances between all loci derived from the final output structure of CBCR and *D* is the set of the wish distances between chromosomal loci. Here, $\bar{d}$ and $\bar{d^{\prime }}$ are the means of and $D^{\prime }$, respectively. In the dSCC formula, rank(·) denotes the rank of a distance, and $\bar{rank(d)}$ and $\bar{rank(d^{\prime })}$ denote mean rank of *D* and $D^{\prime }$ respectively.

In general, the closer the dPCC and dSCC values are to one, the more similar the recreated model is to the actual chromosome. Also, the closer the dRMSE between the actual and recreated chromosome is to zero, the more similar the two structures are. In some instances, such as when the Hi-C data is generated through simulations, these pairwise distances in *D* are known explicitly. In other instances, such as when the Hi-C data is collected from real chromosomes, the actual distances are unknown. In this case, we let *D* be the set of wish distances converted from the input contact frequencies found by (1) and compare these values to $D^{\prime }$.

### 2.8. Hyperparameters

Here, we give a concise list of each hyperparameters used in the evaluation of CBCR. We detail their meaning, their default value (if applicable), and the rational for this choice of default.

- $convg_{0}$: A convergence threshold for inter-curricula dSCC. If the absolute difference between two consecutive sub-curricula dSCC is less than this value, then all of the remaining data is merged together and utilized for one final training. The default value for this hyperparameter is $10^{-6}$ as guided by the findings in [11].
- $convg_{1}$: A convergence threshold for intra-curriculum optimization. If the $l_{2}$ norm of the distance gradients at iteration *t* is less than $conv_{1}$ times the loss at iteration *t*, then we stop optimizing over the current currriculum and move to the next curriculum. The default value for this hyperparameter is $10^{-6}$ as guided by the findings in [11].
- $max\_iter_{0}$: The maximum total number of iterations over all sub-curricula combined. This value bounds the number of iterations that CBCR may run during training. This value is set by the user and should be guided by the size of the input data. If the input data is large, then more iterations will be necessary to optimize sufficiently. Our analysis shows that a value of 4500 is sufficiently large for 100 kb resolution Hi-C data (see Section 3.3).
- $max\_iter_{1}$: The maximum total number of iterations over the final training of CBCR. Recall that if $convg_{0}$ is met, we lump all of the remaining data and use it for one final training. The hyperparameter $max\_iter_{1}$ dictates how many iterations we allow over this final, lump-sum curriculum. This value is separate from $max\_iter_{0}$ in order to ensure that if convergence is met, we do not excessively iterate over the remaining data. For example, if one sets $max\_iter_{0}$ to 10,000, but $convg_{0}$ is met at iteration 1000, the remaining 9000 iterations may be needlessly excessive to meet convergence over the remaining data. Thus, by allowing $max\_iter_{0}$ to a lower, pre-set number, we ensure that the remaining data is still optimized with a reasonable bound of iterations. Like $max\_iter_{0}$, this value is set by the user and should be guided by the size of the input data. Our analysis shows that a value of 500 is sufficiently large for 100 kb resolution Hi-C data (see Section 3.3).
- λ: The learning rate. As we utilize adaptive optimizers, the convergence of CBCR is less sensitive to the initial choice of λ. So long as we choose a value that is sufficiently large, the optimizers will reduce λ as time progresses to ensure convergence. The default value for this hyperparameter is 0.2 as guided by the findings in [11].
- γ: The conversion factor that dictates the relationship between interaction frequency and wish distance given by Equation (2). This can either be a single value, or a range set by the user. For our experiments, we run CBCR for each γ in the set {0.1,0.2,⋯,2.0} and choose the value that maximizes dSCC as the optimal conversion factor. The choice to do this was guided by the findings in [11,37].
- $\gamma_{1}$: The bias parameter for the first moment estimator in the Adam optimizer. The default value for this hyperparameter is 0.9 as recommended in [22].
- $\gamma_{2}$: The bias parameter for the second moment estimator in the Adam optimizer. The default value for this hyperparameter is 0.999 as recommended in [22].
- α: The scaling parameter for the previously seen data in each curriculum. This hyperparameter dictates how much probabilistic mass we assign to the previously seen data in each curriculum. As α is optimized during the training process, its initial value changes during training. The default value for this hyperparameter is 0.5.
- β: The scaling parameter for the new, untrained data in each curriculum. This value is defined to be β:=1−α throughout training. Thus, the default initial value of β is also 0.5.
- Number of Curricula: The total number of curricula. This value dictates how many curricula we divide our data into. This hyperparameter is the central hyperparameter in CBCR. We show that the reconstruction accuracy of CBCR is robust to the choice of this hyperparameter. We also show that setting the number of curricula to be one fifth of the total number of contacts yields an approximate minimization of the run-time of CBCR. See Section 3.1 for details of these findings. According to these findings, the default value for the number of curricula is $\left\lceil \frac{1}{5}N\right\rceil$ where *N* is the number of contact sites corresponding to the input data.

## 3. Results

### 3.1. Finding the Optimal Number of Curricula

One advantage provided by the curriculum learning strategy we adopt is a reduction in the number of computations required during training. For example, assume we would like to generate a structure from a chromosome with 10,000 contact values. If we restrict our maximum number of iterations to 1,000, then an ordinary distance-restraint algorithm would need to do a total of $10,000\times 1000=10^{9}$ parameter updates. If we divide the contacts into 100 curricula, however, then we would need to do $\sum_{i=1}^{100}i\times 10\times 100=5050\times 1000=5.05\times 10^{6}$ parameter updates.

This relationship may seem to imply that the optimal number of curricula is the total number of contacts as this value minimizes the area under the curve. This is not the case due to the relationship between the number of curricula and the rate of convergence of the distance-restraint algorithm which we will now describe. Figure 2 displays the evolution of dSCC over the number of parameter updates (iterations) to elucidate the convergence patterns of CBCR under different values for the number of curricula when evaluated on the yeast data. From this figure, it is clear that the addition of curricula increases the rate of convergence of the distance-restraint algorithm. This relationship likely holds because CBCR retrains previously trained structures as more data is introduced to the training set. This retraining refines the most important components of the structure multiple times, thereby increasing the rate of convergence to the true structure.

Note that, from this figure, it may seem that the rate of convergence is maximized for 600 curricula due to the fact that the dSCC associated with this value begins to increase before the other values. Although this is true, the overall rate of this increase is slower than the value for 100 curricula. This is displayed by the fact that the curve associated with 100 curricula increases sharply after 2000 iterations followed by a sharp flattening shortly thereafter, whereas the curve associated with 600 curricula increases steadily and flattens steadily as well. Thus, although dSCC increases sooner for the 600 curricula trial, the fact that this increase is slower than that of the 100 curricula trial results in the 600 curricula trial reaching the maximum dSCC after the 100 curricula trial. Thus, the convergence of the 600 begins before the 100 curricula trial but reaches convergence slower.

The sooner but slower convergence of the 600 trial can likely be explained by the relationship between the rate at which previously seen data is utilized for retraining and the rate at which we introduce new, unseen data to the training set. For higher values of the number of curricula, the amount of data being introduced per curriculum is smaller than that of lower curriculum values, but the frequency of retraining using previously seen data is higher. This higher rate of retraining using previously seen data leads to a sooner increase in dSCC. The fact that new data is introduced at a lesser rate, however, leads to the slower rate of convergence relative to lower curricula. This relationship implies that there is some optimal choice for the number of curricula that balances the rate of retraining using previously seen data with the rate of introducing new data to the training set. In order to find the approximate optimal choice of the number of curricula, we evaluated the time performance of CBCR on the 90% coverage helical data and the artificially generated yeast data for several values for the number of curricula while measuring the time performance as well as the final dSCC for each trial.

The helical and yeast data have a total of 100 and 610 loci, respectively. To test the time performance of CBCR at various curricula numbers, we evaluated the average time duration and dSCC for both data sets over 30 trials. We divided the helical data into curricula numbers beginning at 5 and increasing to 100 by increments of 5. We divided the yeast data beginning at 10 and increasing by 10 up to 400, and then increasing by 50 up to 600. To maintain experimental consistency, we fixed the maximum total number of iterations to 4500 and the final convergence iteration number to 500. Moreover, we let the maximum number of sub-curricula SCC evaluations be 20 for each run of CBCR.

**Figure 3:**
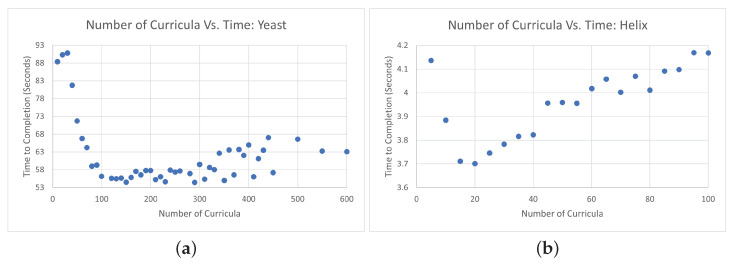
(**a**) Average run time of CBCR for various numbers of curricula evaluated on the yeast data over 30 trials. (**b**) Average run time of CBCR for various numbers of curricula evaluated on the helical data over 30 trials.

Figure 3 depicts the run time of CBCR for various numbers of curricula. There is a notable decrease in the run time for initial increases in the number of curricula, but the run time begins to increase again after a certain number of curricula. This is due to the sooner but slower convergence relationship described above. According to these trials, it seems that this optimal value is about one fifth of the total number of genomic loci being evaluated ($\frac{1}{5}N$).

**Figure 4:**
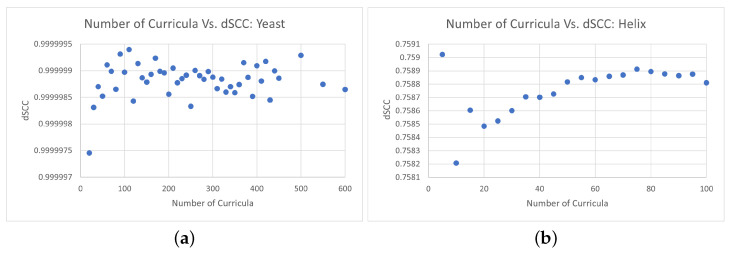
(**a**) Variation in average dSCC for various numbers of curricula evaluated on the yeast data over 30 trials. (**b**) Variation in average dSCC for various numbers of curricula evaluated on the helical data over 30 trials.

It is also important to validate whether the decreased run time of CBCR caused by alterations of the number of curricula comes at the cost of decreased reconstruction accuracy. Figure 4 shows how the average dSCC varies over the number of curricula for 30 trial runs of CBCR. Although there is some variation to the dSCC, the maximum difference for the yeast data is within $10^{-7}$ and the maximum difference for the helix data is within $10^{-4}$. This suggests that, although there is some variation in the dSCC when different numbers of curricula are utilized, the reconstructive performance of CBCR is somewhat robust to this specific hyperparameter, especially when evaluated on larger inputs.

### 3.2. Time Performance

**Figure 5:**
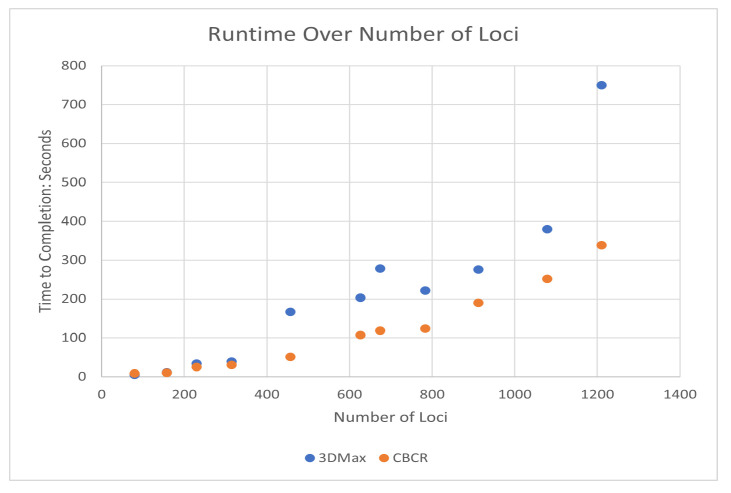
Time comparison between CBCR and 3DMax for various numbers of loci.

To test whether the decrease in run time associated with the addition of curricula is substantial, we compared the time performance of CBCR with 3DMax [11]. We chose 3DMax in this comparison due to the fact that both 3DMax and CBCR are distance-restraint algorithms that rely on gradient ascent using Adagrad and similar, likelihood-based objective functions that were both implemented in MATLAB. We tested both CBCR and 3DMax on several chromosomes in the GM12878 cell line using the same learning rate, convergence thresholds, and maximum number of iterations. For CBCR, we chose the number of curricula to be one fifth of the number contacts for each chromosome. Figure 5 shows the relationship between run-time and number of loci in the interaction frequency matrix for 3DMax and CBCR. Specifically, each data point corresponds to the average time duration of 15 runs for each algorithm. From this figure, it is clear that the addition of curricula does not lead to a substantial increase in time performance for low loci values, but there is a significant increase in time performance for larger loci values. This suggests that the addition of curricula decreases the computational load of our distance-restraint algorithm and that this decrease is larger for higher dimensional data. All tests were run on an HP Pavillion x360 with an Intel Core i7-8550U CPU at 1.99 GHz and 8 gb of RAM.

### 3.3. Reconstruction Accuracy: Real Data

**Figure 6:**
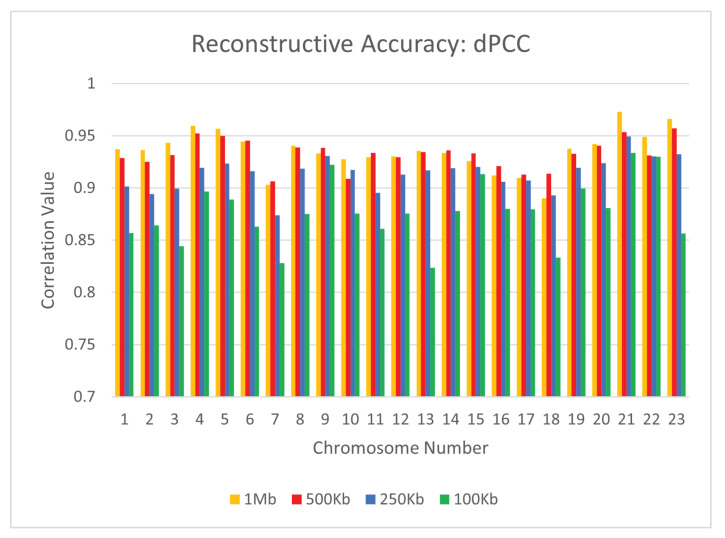
Comparison of distance Pearson Correlation Coefficient (dPCC) for CBCR on all 23 chromosomes at four different resolutions: 1 mb, 500 kb, 250 kb, and 100 kb.

**Figure 7:**
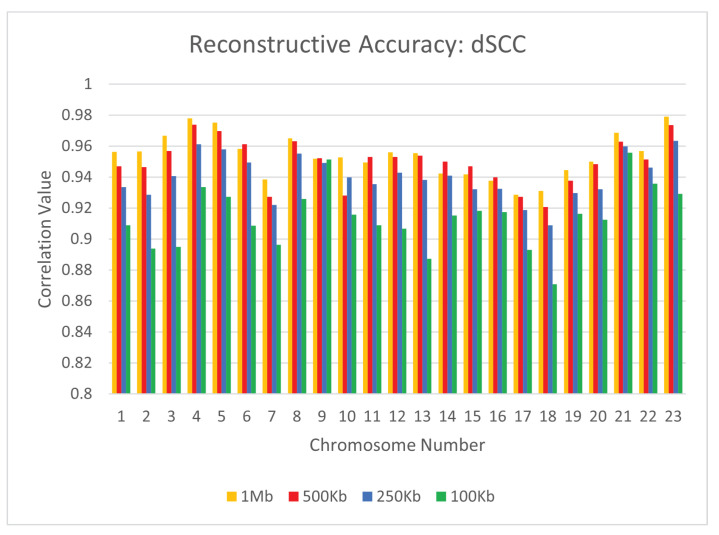
Comparison of dSCC for CBCR on all 23 chromosomes at the four different resolutions.

**Figure 8:**
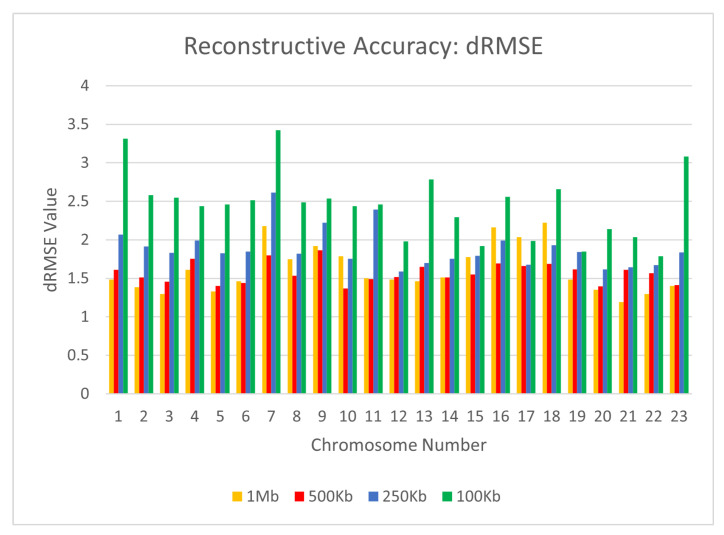
Comparison of dRMSE for CBCR on all 23 chromosomes at the four different resolutions.

To test the reconstructive performance of CBCR on real data, we evaluated the dSCC, dPCC, and dRMSE of our outputs when evaluated on the GM12878 cell line with the Mbol restriction enzyme. For the following experiments, we utilized the primary-replicate combined mappings as the input. In order to account for the variation in size of the Hi-C matrices in this data set, we once again chose the number of curricula to be one fifth of the total number of loci for every chromosome. We also utilized a maximum number of iterations of 10,000. Specifically, we used $max\_iter_{0}=9000$ and $max\_iter_{1}=1000$. We evaluated the performance of CBCR at four different resolutions: 1 mb, 500 kb, 250 kb, and 100 kb on all 23 chromosomes. Figure 6, Figure 7 and Figure 8 show the effect of the resolution of the input on the reconstructive performance of CBCR.

From these figures, one can tell that the performance of CBCR reduces with an increase in resolution. This is typical for most algorithms due to the fact that more data results in a higher accumulation of error, thereby reducing reconstruction accuracy.

**Figure 9:**
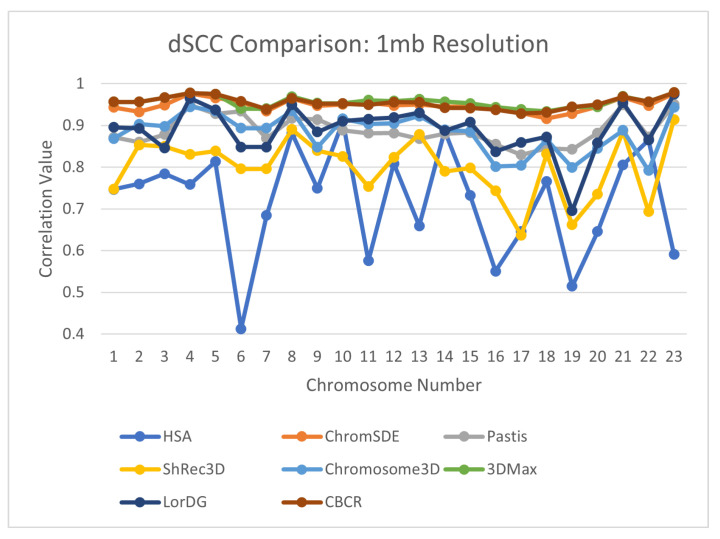
Comparison of dSCC between CBCR and 7 other methods on chromosomes 1–23 at 1 mb resolution.

**Figure 10:**
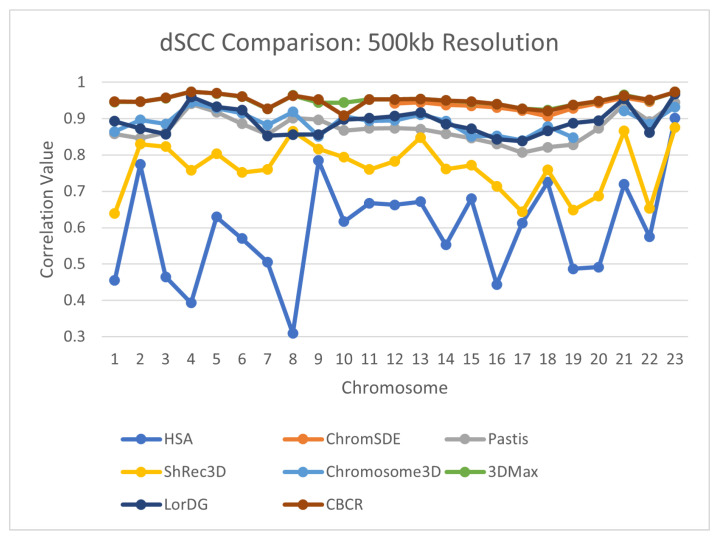
Comparison of dSCC between CBCR and 7 other methods on chromosomes 1–23 at 500 kb resolution.

**Figure 11:**
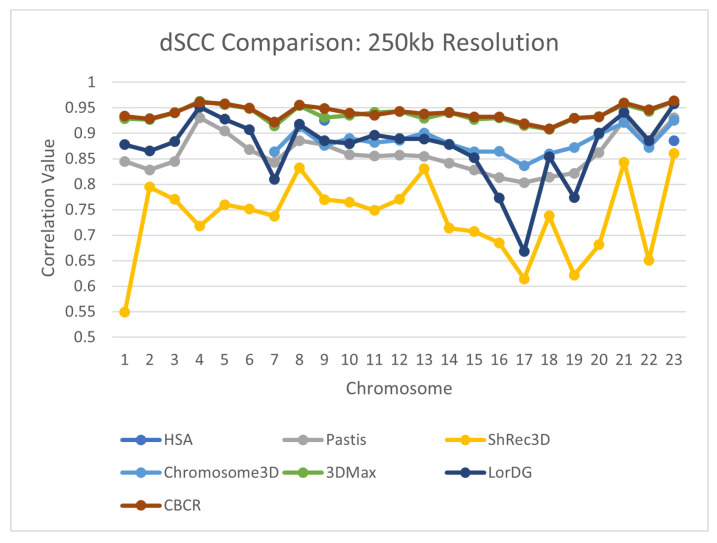
Comparison of dSCC between CBCR and other methods on chromosomes 1–23 at 250 kb resolution.

**Figure 12:**
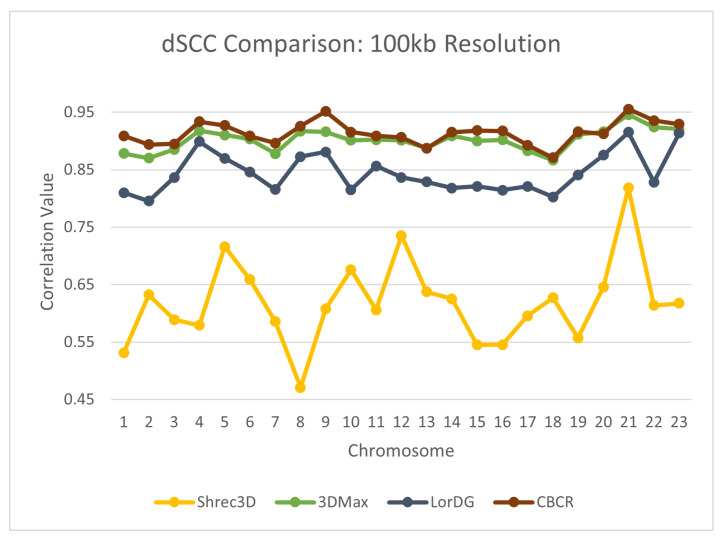
Comparison of dSCC between CBCR and other methods on chromosomes 1–23 at 100 kb resolution.

To further evaluate the reconstructive accuracy of CBCR, the dSCC of the generated structures from each chromosome were compared to the dSCC of structures generated by seven other methods—Shrec3D [10], 3DMax [11], Chromosome3D [13], LorDG [20], ChromSDE [12], HSA [23], Pastis [38]. The models from the methods with which CBCR was compared came from the GSDB database [26]. The models from the GSDB database were fit using multiple hyperparameters, and the best models were selected for publication. The results of this comparison are shown in Figure 9, Figure 10, Figure 11 and Figure 12. Note that several values for existing methods are absent, especially in the high resolution data. This is due to the fact that most methods do not perform on higher resolution data due to computational constraints.

From these figures, one can see that a drop in reconstruction accuracy with an increase in resolution is common amongst all algorithms in comparison. This drop for CBCR, however, is less when compared to all other algorithms. Due to this fact, CBCR outperforms all other algorithms in the comparison for the 100 kb resolution data in terms of reconstruction accuracy. This suggests that the use of curricula increases the robustness of CBCR to increases in resolution.

## 4. Validation

Although CBCR can reproduce wish distances with a high degree of accuracy, these wish distances are merely an estimate of the actual distances associated with the 3D structure of the input chromosome. In order to verify that the outputs of CBCR reflect the true 3D structure of the input chromosome, we validated our results with data obtained from independent biochemical assays such as the ChIA-PET, HiChIP, and FISH data.

### 4.1. Reproducibility Across Hi-C Experiments: Primary vs. Replicate

**Figure 13:**
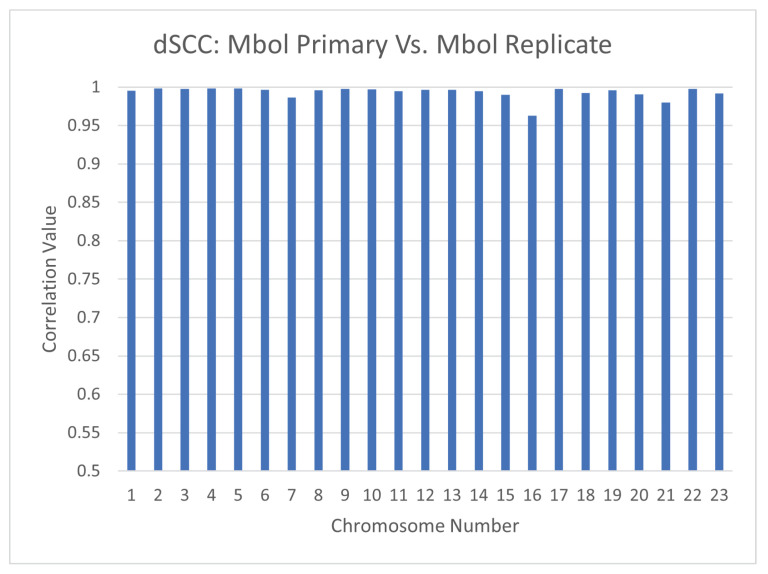
dSCC computed between the outputs of CBCR from the primary and replicate mappings with the Mbol restriction enzyme at 1mb resolution.

**Figure 14:**
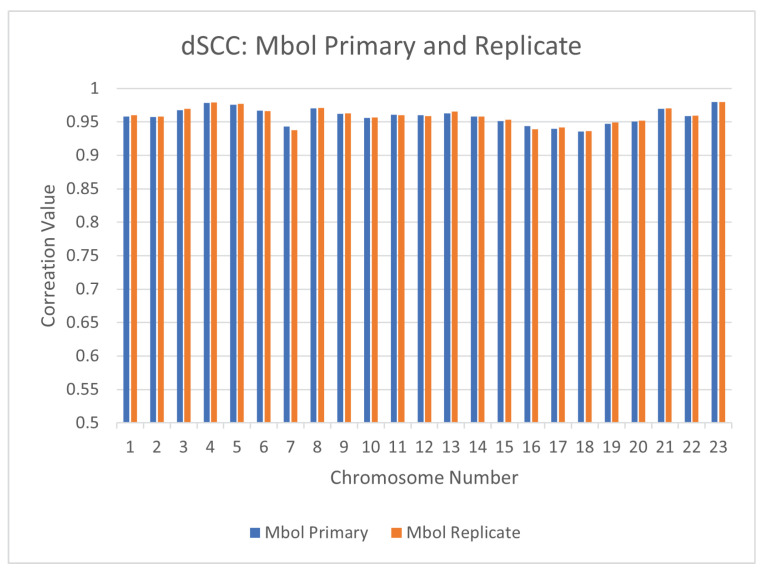
Comparison of the output dSCC for CBCR utilizing the primary and replicate mappings with the Mbol restriction enzyme at 1mb resolution.

**Figure A1:**
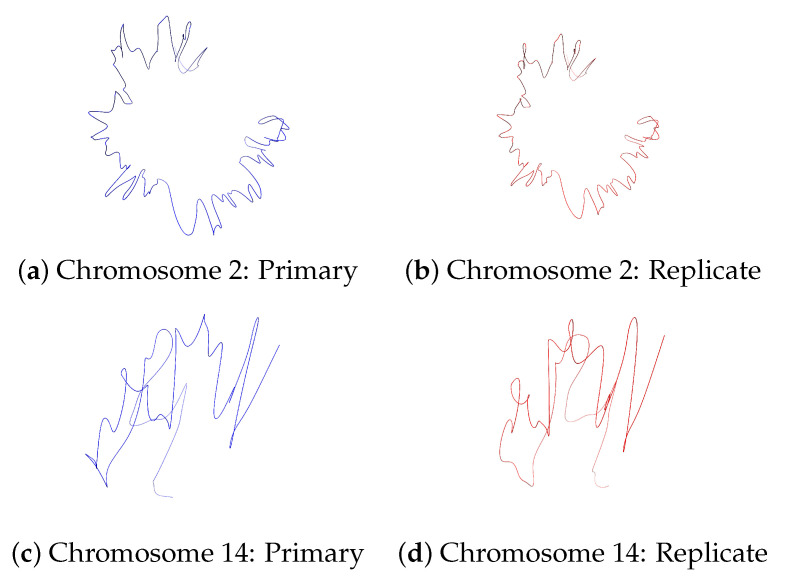

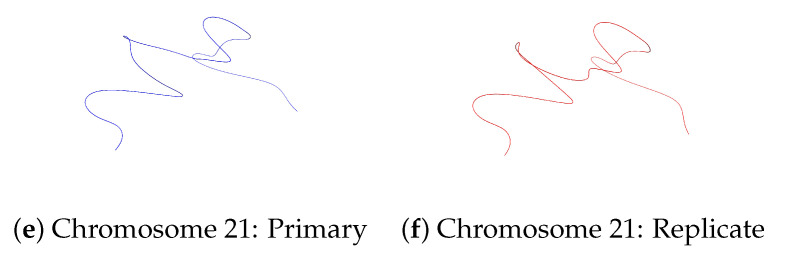
3D structures produced by CBCR on the primary and replicate maps of GM12878 at 1 mb.

To ensure the reproducibility of CBCR across different Hi-C experiments, we also ran the same experiments as above on the primary and replicate mappings at 1mb resolution separately and computed the dSCC between their respective outputs. Figure 13 shows the results of this test. From this figure, it is clear the the dSCC between the primary and replicate structures is close to one for all chromosomes, thereby implying that CBCR can replicate results across separate Hi-C experiments. We also computed the dSCC between the outputs of CBCR and the wish distances of the corresponding input for the primary and replicate mappings in order to ensure that CBCR maintains the same reconstructive accuracy regardless of the mapping choice. Indeed, Figure 14 shows that the reconstructive accuracy of CBCR is similar across each of these mappings. We provide visualizations of these structures in Appendix C (Figure A1) to exhibit the qualitative comparability between the primary and replicate results as well.

### 4.2. Reproducibility Across Restriction Enzymes: Mbol vs. DPnII

**Figure 15:**
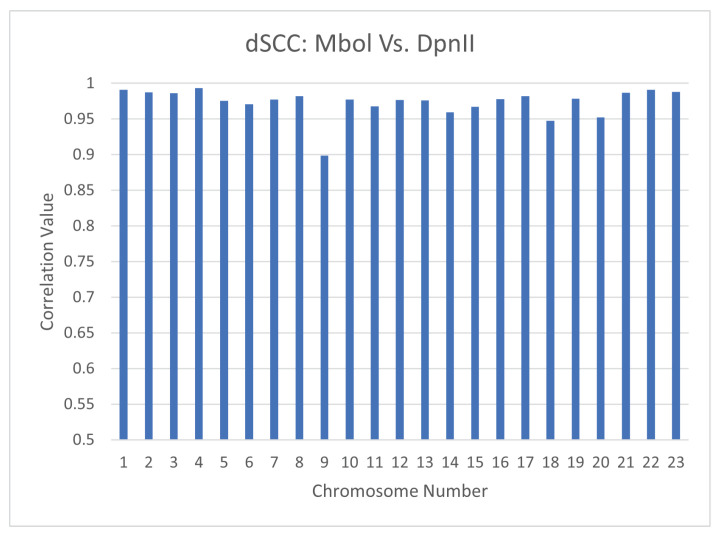
dSCC computed between the outputs of CBCR from the Mbol and DpnII restriction enzymes at 1mb resolution.

**Figure 16:**
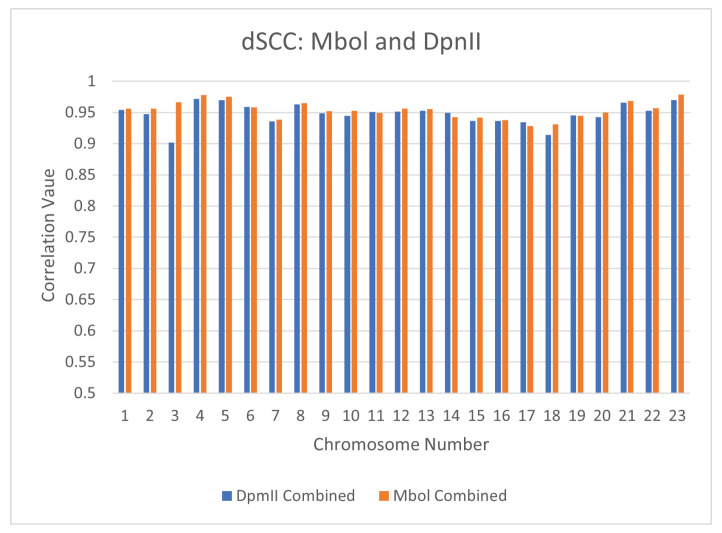
Comparison of the output dSCC for CBCR utilizing the Mbol and DpnII restriction enzymes at 1mb resolution.

**Figure A2:**
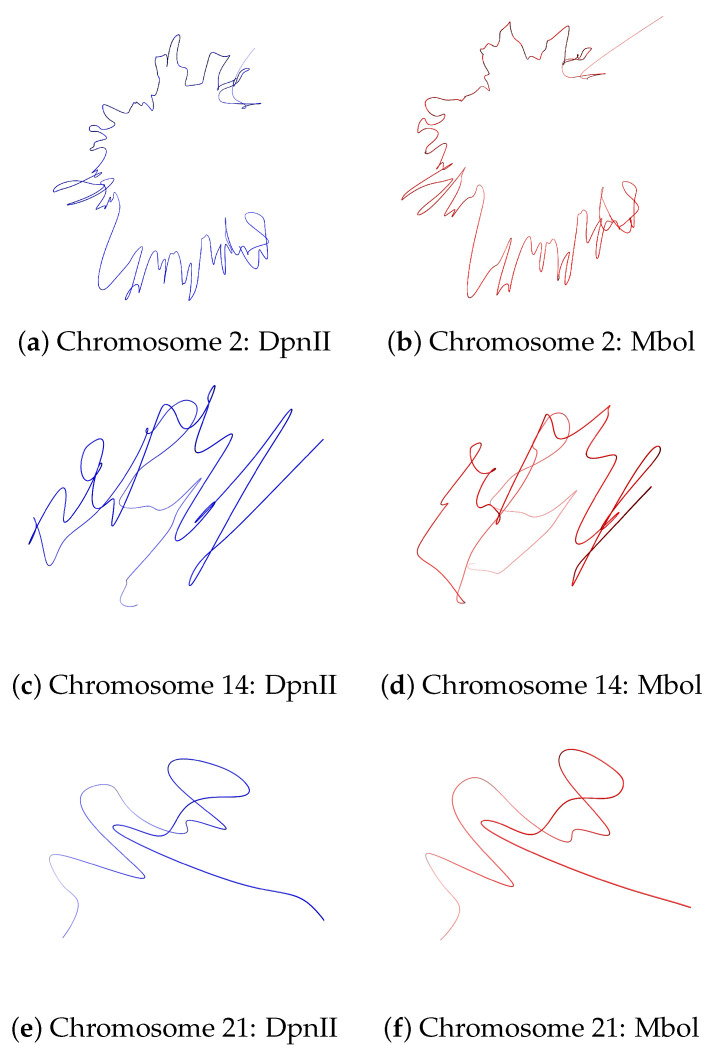
3D structures produced by CBCR on the DpbII and Mbol restricted maps of GM12878 at 1 mb.

The choice of restriction enzyme may influence the results of a Hi-C experiment. So far, our analysis of the performance of CBCR on real data has been restricted to Hi-C experiments performed utilizing the Mbol restriction enzyme. To ensure that CBCR reproduces similar outputs regardless of the choice of restriction enzyme, we generated structures utilizing inputs from Mbol restricted experiments and DpnII restricted experiments separately. All models were generated using 1mb resolution of the primary-replicate combined maps. We then calculated the dSCC between the Mbol and DpnII outputs. Figure 15 shows the results of this test. The majority of the correlation values are greater than 0.95, thereby implying that CBCR produces similar structures regardless of the choice of restriction enzyme. We also computed the dSCC between the outputs of CBCR and the wish distances of the corresponding input for the Mbol and DpnII inputs in order to ensure that CBCR maintains the same reconstructive accuracy regardless of the restriction enzyme choice. Figure 16 shows that, with the exception of chromosome 3, the reconstructive accuracy of CBCR is similar regardless of the choice of restriction enzyme. We provide visualizations of these structures in Appendix C (Figure A2) to exhibit the qualitative comparability between the primary and replicate results as well.

### 4.3. Validation on FISH Data

To further validate the outputs of CBCR, we compared the distances associated with models generated by CBCR to the actual distances in the underlying chromosome measured using FISH. To do this, we used the FISH distance data from the GM12878 cell line provided by Rao et al. [25]. In the study conducted by Rao et al. two peaked regions, L1 and L2, were selected from chromosomes 11, 13, 14, and 17 using their HiCCUPS loop detection algorithm. A third, control region, L3, was also selected from each of these chromosomes as well. The authors then found the distances between regions L1 and L2 as well as the distances between L2 and L3 using 3D FISH for each chromosome. For each chromosome, the distance between L1 and L2 was shorter than the distance between L2 and L3. Thus, we would like to confirm that this relationship holds for the outputs of CBCR as well.

To validate CBCR on this FISH data, we first identified the loci that exist in regions L1, L2 and L3 for the 250 kb and 100 kb resolution Hi-C data. We chose these resolutions because the 500 kb and 1 mb resolutions are not fine enough to accurately fit within the regions evaluated in the FISH data. Once these loci were identified, we then computed the distance between these regions using the outputs of CBCR at these resolutions. Table 1 and Table 2 show the distances between these regions for the outputs of CBCR for 250 kb and 100 kb resolution data. From these tables, it is clear that the distance between the L1 and L2 regions is shorter than the distance between the L2 and L3 regions for each chromosome at both resolutions as desired.

### 4.4. Valitation on ChIA-PET and HiChIP Data

ChIA-PET and HiChIP data measure interaction frequencies between specific proteins in the chromosome. From these interaction frequencies, it is possible to identify loops in the structure of the chromosome that are created by these protein specific interactions. In this study, we used publicly available HiChIP data that measures genome wide interactions mediated by the H3K27ac protein [30,31,32], and publicly available ChIA-PET data mediated by the RNAPolII protein from the NCBI GEO database (GEO accession: GSE72816) [39]. Both of these data sets were derived from the GM12878 cell line. We utilized both of these data sets to validate the outputs of CBCR. Since CBCR relies on cis-chromosomal contacts, we only considered the cis-chromosomal interactions from these data sets. Our validation relied on splitting both data sets into two groups: loops and non-loops. Loops were identified from the ChIA-PET data by considering genomic regions that had a PET interaction frequency greater than or equal to three. Loops were identified from the HiChIP data using the peak calling algorithm, HiCCUPS, introduced in Rao et al. [25]. Once these looped and non-looped regions were identified and separated, we computed the distance between the corresponding loci from the models generated by CBCR at 100 kb resolution in both the looped and non-looped sets. If the models generated by CBCR are indeed representative of the true chromosome structure, the distances between looped regions should be lower than the distances between non-looped regions.

**Figure 17:**
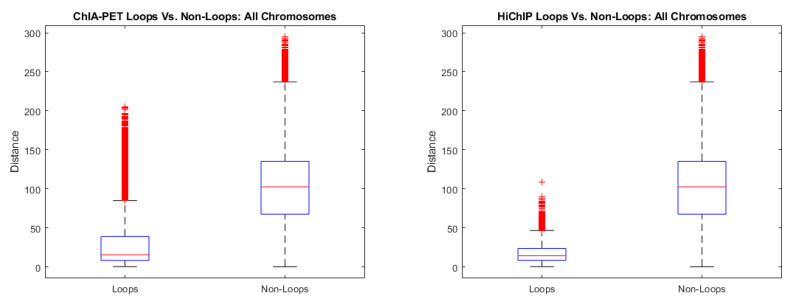
(**a**) Box plots comparing distances within looped regions to distances within non-looped regions for all chromosomes combined identified by the ChIA-PET data. (**b**) Box plots comparing distances within looped regions to distances within non-looped regions for all chromosomes combined identified by the HiChIP data.

Figure 17 shows the box plots for the distances calculated from CBCR’s outputs at 100 kb resolution between looped and non-looped regions across all chromosomes combined for the ChIA-PET and HiChIP data. From these plots, it is clear that looped regions do indeed have shorter distances than non-looped in the models generated by CBCR. Moreover, this relationship holds across all chromosomes individually as well. See Appendix C for the figures illustrating these comparisons.

### 4.5. Comparison of Outputs Between CBCR and 3DMax

**Figure 18:**
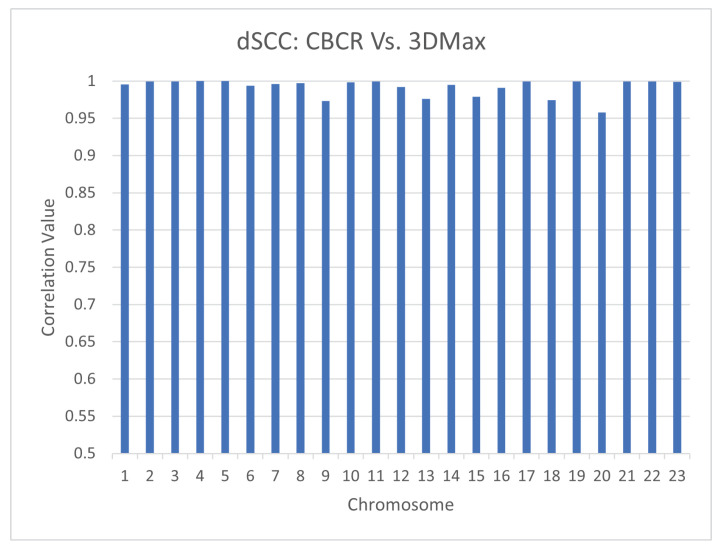
dSCC calculated between the outputs of CBCR and the outputs of 3DMax on GM12878 at 100 kb resolution.

Although CBCR is a specific algorithm, curriculum learning is a very general training strategy. CBCR is a particular application of curriculum learning to a distance restraint training algorithm that was inspired by 3DMax [11]. For this reason, the output structures of CBCR are similar to the output structures of 3DMax. Figure 18 shows the dSCC values between the outputs of CBCR and the outputs of 3DMax on GM12878 at 100 kb resolution. Since the correlation values are all above 0.95, it is clear that the structures are indeed similar.

We have seen that the use of curricula increases the time performance of our training algorithm and also increases the algorithm’s robustness to increases in resolution of the input data. Thus, the similarity between the outputs of CBCR and the outputs of 3DMax seems to imply that the adaptation of distant restraint algorithms to a curriculum-tailored learning strategy may be an effective way to increase the performance of the method while preserving the general nature of its outputs. This preservation of the general nature of the output structures is important because distant-restraint algorithms often rely on heuristics guided by the assumed behavior of the input chromosomes [9]. Thus, if curriculum learning does indeed preserve the nature of the outputs of distance-restraint algorithms, then distance-restraint algorithms may be adapted to a curriculum-tailored approach in order to increase their performance without altering the general behavior of the non-adapted distance-restraint algorithm. To further justify that this is likely the case in the context of CBCR and 3DMax, we compared their validations on HiChIP and ChIA-PET data as performed in Section 4.4.

**Figure 19:**
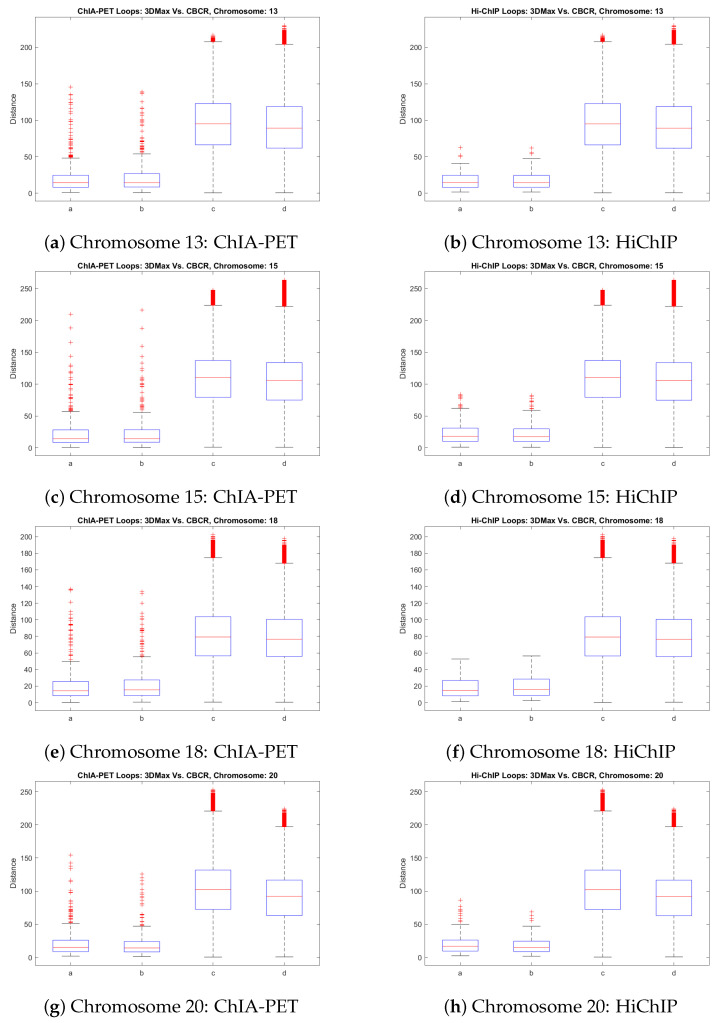
Comparison of the loop and non-loop model distances between CBCR and 3DMax at 100 kb for the HiChIP and ChIA-PET data. The first and third boxes in each plot correspond to CBCR and the second and fourth boxes correspond to 3DMax.

Figure 19 shows the comparison of looped and non-lopped distances between the four least similar outputs of CBCR and 3DMax on GM12878 at 100 kb resolution using the same data in Section 4.4. We consider the least similar structures to be those who have the lowest dSCC in Figure 16: chromosomes 13, 15, 18, 20. Note that due to variability in the 3D outputs between CBCR and 3DMax, the structures must first be scaled to each other before making meaningful comparisons between their output structure distances. This scaling was performed using standard procrustes analysis. From these figures, it is clear that CBCR and 3DMax have similar performance on both the ChIA-PET and HiChIP data. The loops plots for both methods show that the 3D distances across the loop positions are consistently closer to each other compared to those on the non-loops. Also, that the non-loops plots is much higher than the loop plots for both methods suggests the differences between the two groups.

## 5. Discussion

In this paper, we presented a novel technique for chromosome structure reconstruction based on curriculum learning called CBCR. CBCR is a distance-restraint method that trains on subsets of Hi-C data that increase in difficulty in order maximize a scaled log likelihood objective function utilizing gradient ascent. The results of CBCR on real Hi-C data show that CBCR performs better than most existing methods. In addition to these findings, we show that the use of curricula aids in the time performance of CBCR by both reducing the computational cost and accelerating the convergence of the distance-restraint algorithm. Also, CBCR is more robust to increases in Hi-C resolution when compared with other similar methods. This increased robustness yields a superior reconstruction accuracy of CBCR for higher resolution Hi-C data compared to these other methods. Finally, we showed that CBCR produces accurate results when validated on FISH, HiChIP, and ChIA-PET data as well.

The increases in reconstructive performance of CBCR are attributed to the continual retraining of the predicted structure using the most important contacts underlying the 3D structure of the chromosome. The increase in the time performance is due to the fact that data is iteratively added to prevent redundant iterations over data that is not relevant to the structure of the chromosome. Although we utilize an objective function that is tailored to curriculum learning, the basic idea of curriculum learning can be applied on top of any distance-restraint algorithm. Curriculum training may prove to be an effective way to increase the rate of convergence of standard distance-restraint algorithms while decreasing their total computational load, thereby allowing distance-restraint algorithms to be implemented on higher resolution input data while maintaining reconstructive performance and decreasing run time. Moreover, the curriculum approach opens opportunities to modify hyperparameters in the objective function, such as α, $p_{k}$, and $q_{k}$, that allow for scaling of certain data. These aspects of curriculum learning are very general and can potentially be applied to many distance-restraint based algorithms. Moreover, it may be possible for the curricula to be divided based upon a different metric than contact frequency. These are all potential ways in which curriculum learning may continue to improve the performance of chromosome reconstruction algorithms.

## Figures and Tables

**Table 1:** Distances between fluorescent in situ hybridization (FISH) regions computed from CBCR models at 250 kb resolution.

Chromosome	L1-L2 Distance	L2-L3 Distance
11	15.8	17.2
13	5.7	21.5
14	15.7	27.5
17	7	40.2

**Table 2:** Distances between FISH regions computed from CBCR models at 100 kb resolution.

Chromosome	L1-L2 Distance	L2-L3 Distance
11	13.7	29.5
13	8	31
14	28.9	33.4
17	16.2	42.2

**Figure A3:**
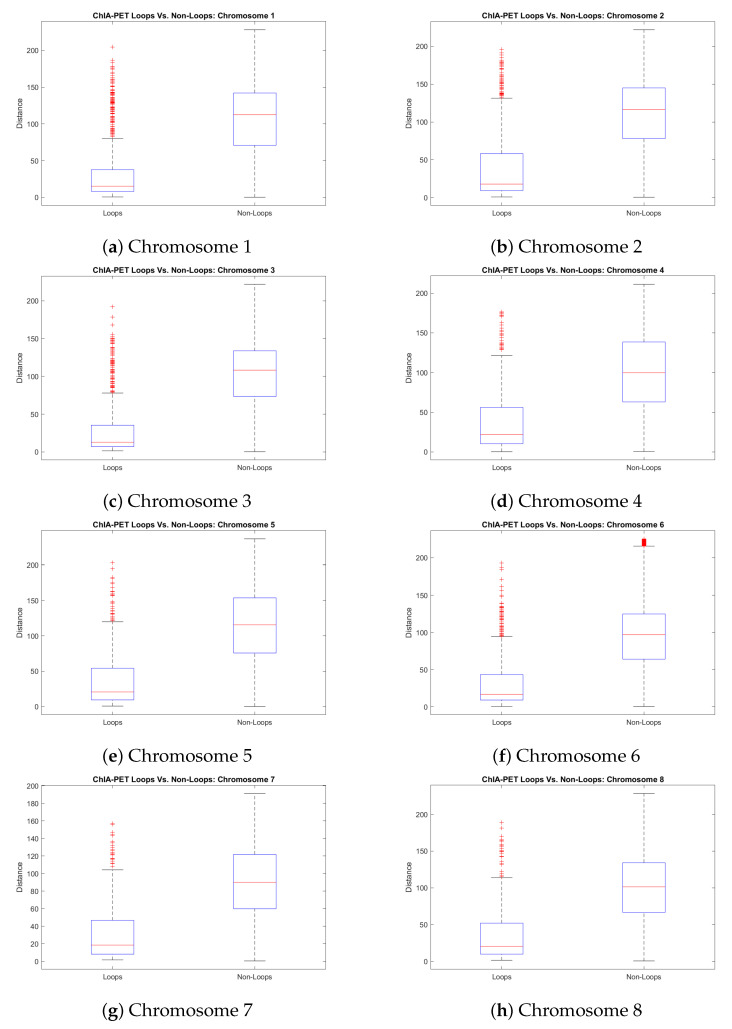

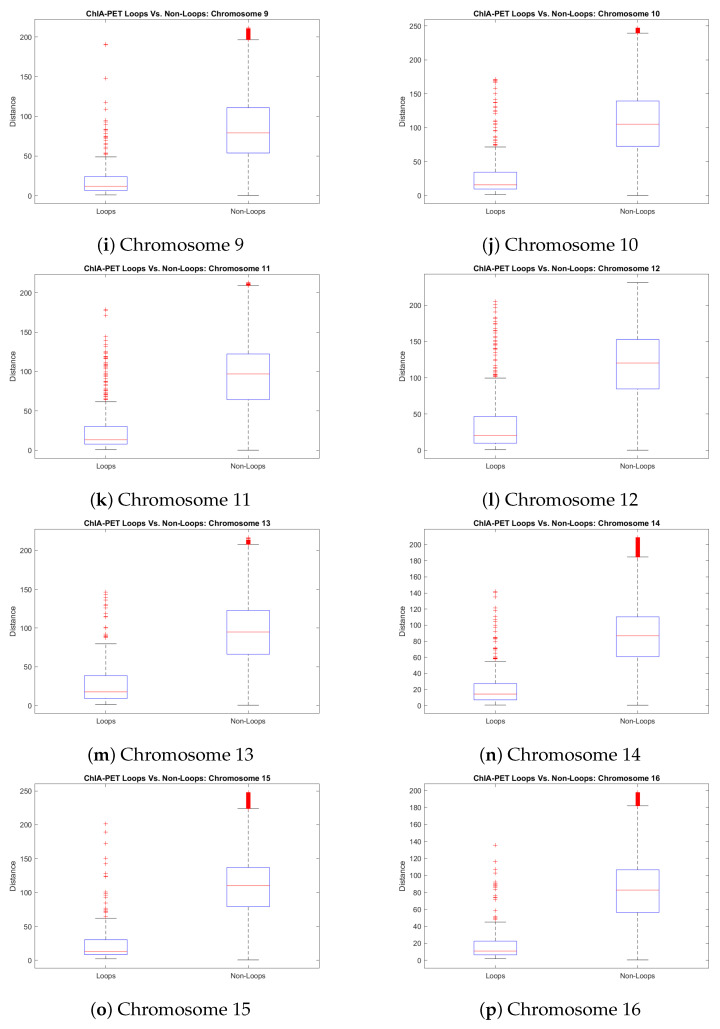

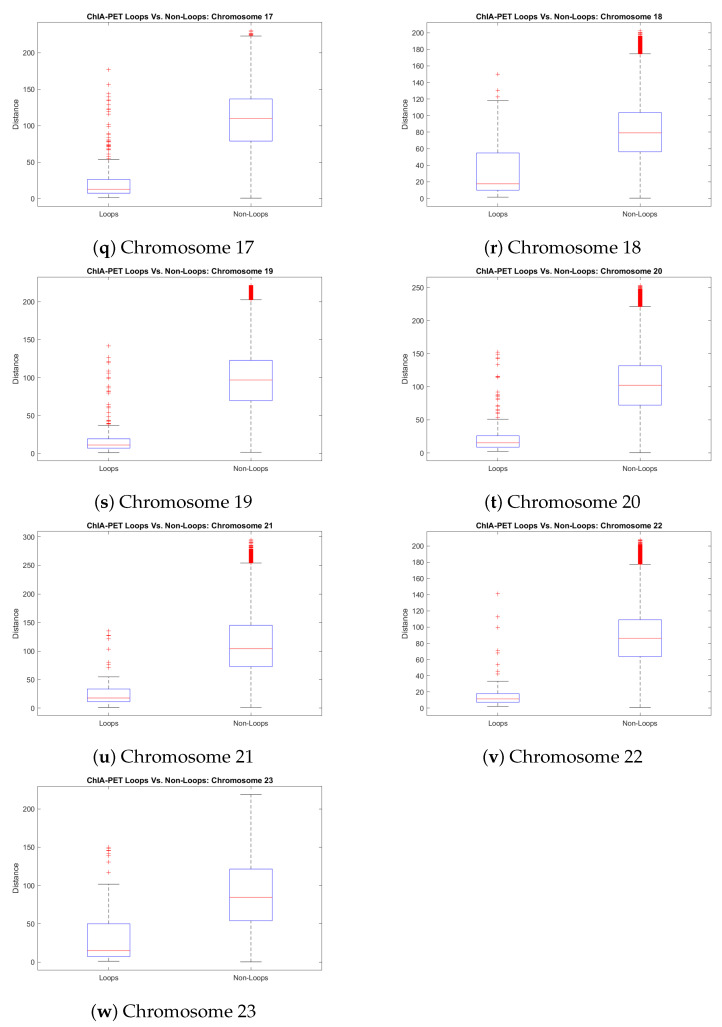
Comparisons of the looped and non-looped distances of CBCR’s outputs at 100kb resolution for the ChIA-PET data.

**Figure A4:**
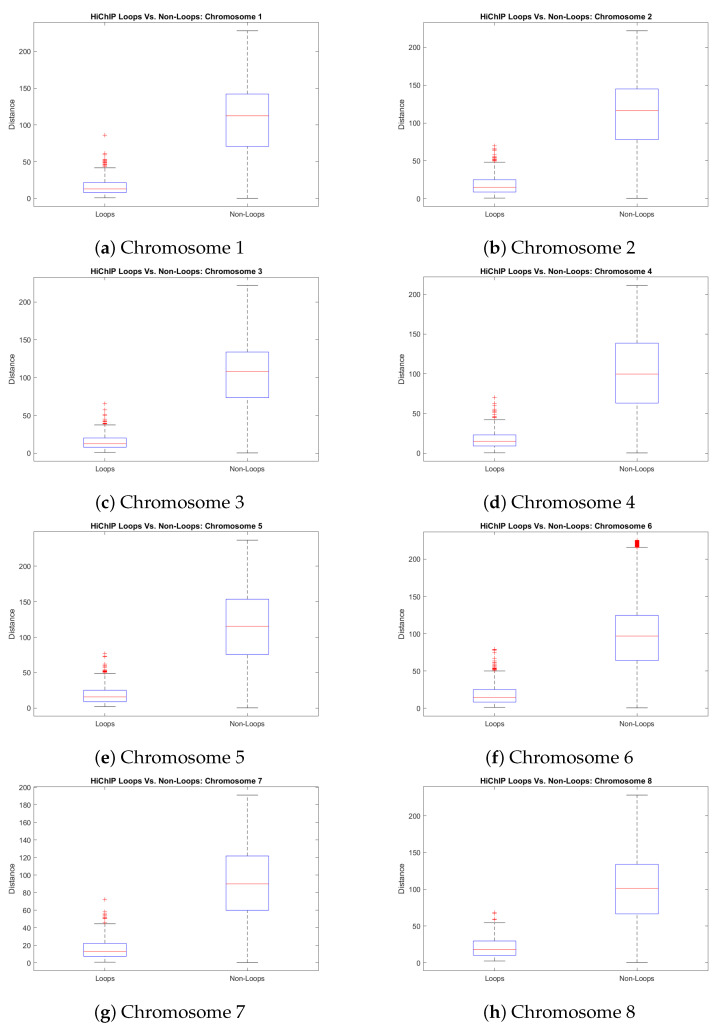

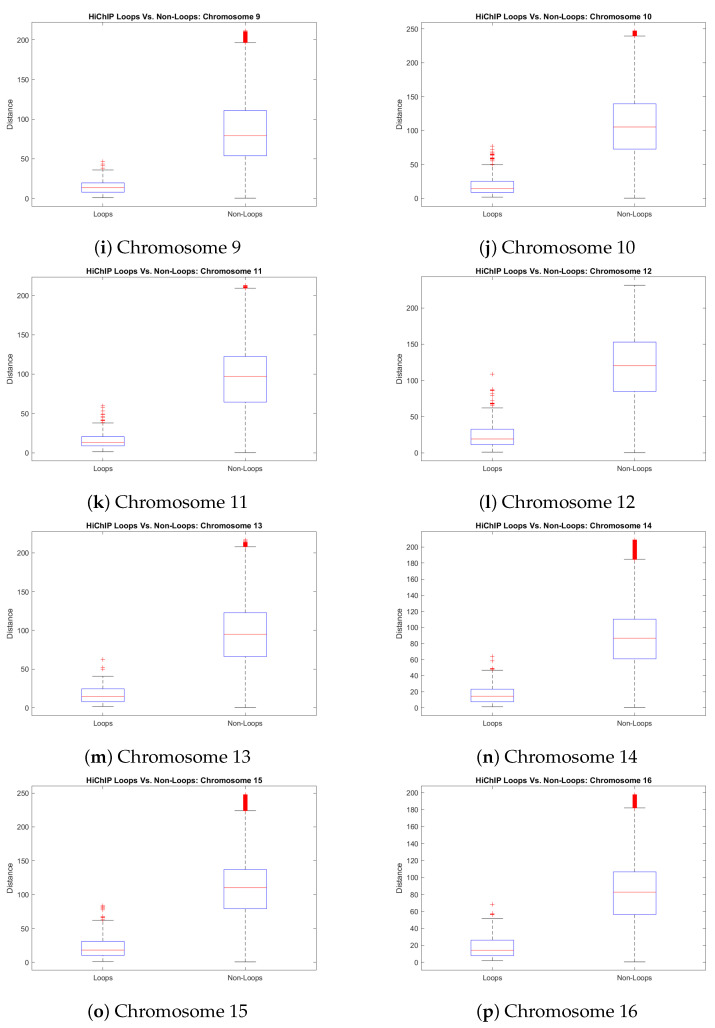

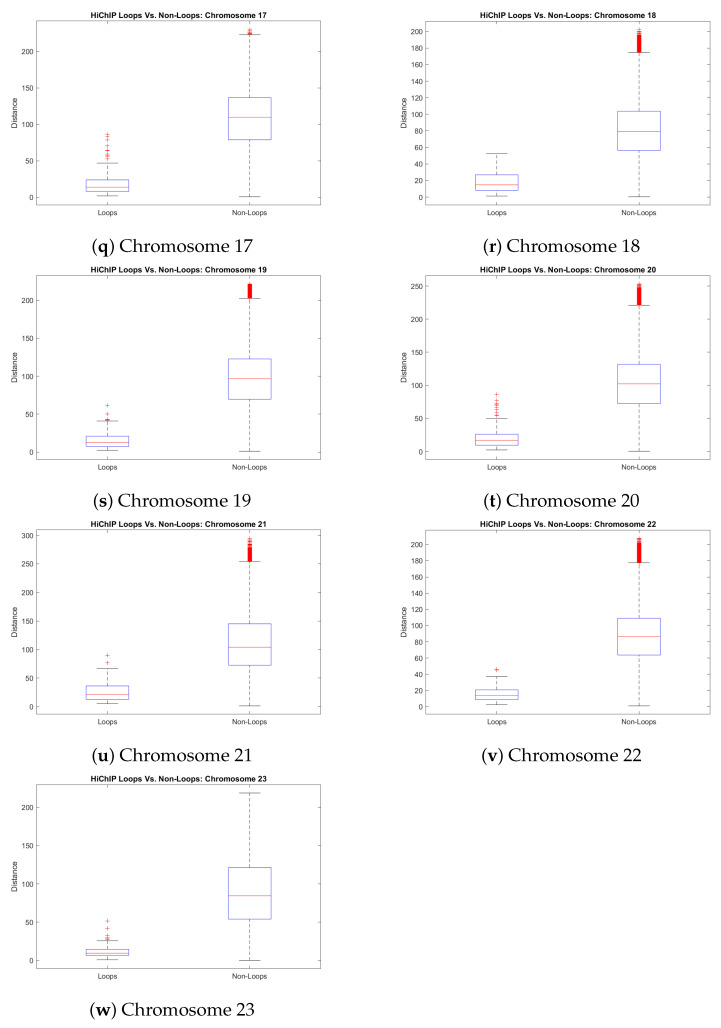
Comparisons of the looped and non-looped distances of CBCR’s outputs at 100kb resolution for the HiChIP Data.

## Data Availability

The code for CBCR as well as the pdb files for our reconstructed structures can be found at https://github.com/OluwadareLab/CBCR.

## Abbreviations

The following abbreviations are used in this manuscript:

3D	Three dimensional
3C	Chromosome Conformation Capture
dSCC	Distance Spearman Correlation Coefficient.
dPCC	Distance Pearson Correlation Coefficient.
dRMSE	Distance Root Mean Squared Error.
CBCR	Curriculum Based Chromosome Reconstruction.
GSDB	Genome Structure Database
FISH	Fluorescence in Situ Hybridization
ChIA-PET	Chromatin Interaction Analysis by Paired-End Tag Sequencing
HiChIP	Hi-C Chromatin Immunoprecipitation
GEO	Gene Expression Omnibus

## Appendix A

### Appendix A.1

To calculate the derivative of log(L(S)) with respect to the *x*, *y*, and *z* coordinates, we utilize the chain rule. If we set

(A1) $$\begin{array}{cc} V & =\alpha p_{k}\sum_{i=1}^{r}{\left({dt\prime }_{i}\left(k\right)-{dt}_{i}\left(k\right)\right)}^{2}+{\beta q}_{k}\sum_{i=1}^{s}{\left({d\prime }_{i}\left(k\right)-d_{i}\left(k\right)\right)}^{2} \end{array}$$

(A2) $$\begin{array}{cc} W & =\sqrt{\frac{V}{r+s}} \end{array}$$

(A3) $$\begin{array}{cc} E & =-(\alpha r+\beta s)\log(W) \end{array}$$

then we have

(A4) $$\log\left(L\left(S\right)\right)=E-\frac{r+s}{2}$$

Therefore, by the chain rule, for some distance $d^{\prime }\in Dt^{\prime }\left(k\right)\cup D^{\prime }\left(k\right)$, using the chain rule, we have

(A5) $$\frac{\partial \log(L(S))}{\partial d^{\prime }}=\frac{\partial E}{\partial W}\cdot \frac{\partial W}{\partial V}\cdot \frac{\partial V}{\partial d^{\prime }}.$$

Taking the respective partial derivatives yields

(A6) $$\begin{array}{cc} \frac{\partial E}{\partial W} & =\frac{-(\alpha r+\beta s)}{W} \end{array}$$

(A7) $$\begin{array}{cc} \frac{\partial W}{\partial V} & =\frac{1}{2\sqrt{(r+s)V}} \end{array}$$

(A8) $$\begin{array}{cc} \frac{\partial V}{\partial d^{\prime }} & =\left\{\begin{array}{cc} 2\alpha p_{k}\left(d^{\prime }\left(k\right)-d\left(k\right)\right) & \mathrm{if}\;d^{\prime }\in D^{\prime }\left(k\right)\\ 2\beta q_{k}\left(dt^{\prime }\left(k\right)-dt\left(k\right)\right) & \mathrm{if}\;d^{\prime }\in Dt^{\prime }\left(k\right) \end{array}\right. \end{array}$$

where $d^{\prime }\left(k\right)$ or $dt^{\prime }\left(k\right)$ are the wish distances corresponding to $d^{\prime }$ depending on whether $d^{\prime }$ is in $D^{\prime }\left(k\right)$ or $Dt^{\prime }\left(k\right)$ respectively. Well, $d^{\prime }=\sqrt{{\left(x_{i}-x_{j}\right)}^{2}+{\left(y_{i}-y_{j}\right)}^{2}+{\left(z_{i}-z_{j}\right)}^{2}}$ where $\left(x_{i},y_{i},z_{i}\right)$ and $\left(x_{j},y_{j},z_{j}\right)$ are the 3D coordinates of locus *i* and *j* respectively that uniquely determine $d^{\prime }$. Therefore, using the chain rule again, the partial derivative of $d^{\prime }$ with respect to a coordinate $c_{i}\in \left\{x_{i},y_{i},z_{i}\right\}$ is given by

(A9) $$\frac{\partial d^{\prime }}{\partial c_{i}}=\frac{c_{i}-c_{j}}{\sqrt{{\left(x_{i}-x_{j}\right)}^{2}+{\left(y_{i}-y_{j}\right)}^{2}+{\left(z_{i}-z_{j}\right)}^{2}}}$$

where $c_{j}\in \left\{x_{j},y_{j},z_{j}\right\}$ is the corresponding element to $c_{i}$ (i.e., if $c_{i}=x_{i}$, then $c_{j}=x_{j}$). Similarly, for $c_{j}\in \left\{x_{j},y_{j},z_{j}\right\}$, we have

(A10) $$\frac{\partial d^{\prime }}{\partial c_{j}}=\frac{c_{j}-c_{i}}{\sqrt{{\left(x_{i}-x_{j}\right)}^{2}+{\left(y_{i}-y_{j}\right)}^{2}+{\left(z_{i}-z_{j}\right)}^{2}}}.$$

We use these equations to calculate the derivative of log(L(S)) with respect to a given spatial parameter *c* corresponding to the distance $d^{\prime }$ given by

(A11) $$\frac{\partial \log(L(S))}{\partial c}=\frac{\partial E}{\partial W}\cdot \frac{\partial W}{\partial V}\cdot \frac{\partial V}{\partial d^{\prime }}\cdot \frac{\partial d^{\prime }}{\partial c}$$

### Appendix A.2

To calculate the derivative of log(L(S)) with respect to α and β we again utilize the chain rule. Since β=1−α, we only need to derive with respect to α. Utilizing the same Equations (A1)–(A3), we have

(A12) $$\frac{\partial \log(L(S))}{\partial \alpha }=\frac{\partial E}{\partial W}\cdot \frac{\partial W}{\partial V}\cdot \frac{\partial V}{\partial \alpha }.$$

Well, $\frac{\partial E}{\partial W}$ and $\frac{\partial W}{\partial V}$ are still given by Equations (A6) and (A7). Since $V=\alpha p_{k}\sum_{i=1}^{r}$${\left({dt\prime }_{i}\left(k\right)-{dt}_{i}\left(k\right)\right)}^{2}+{(1-\alpha )q}_{k}\sum_{i=1}^{s}{\left({d\prime }_{i}\left(k\right)-d_{i}\left(k\right)\right)}^{2}$, the partial derivative of *V* with respect to α is given by

(A13) $$\frac{\partial V}{\partial \alpha }=p_{k}\sum_{i=1}^{r}{\left({dt\prime }_{i}\left(k\right)-{dt}_{i}\left(k\right)\right)}^{2}-q_{k}\sum_{i=1}^{s}{\left({d\prime }_{i}\left(k\right)-d_{i}\left(k\right)\right)}^{2}$$

## Appendix B

We use the number of curricula and $max\_iter_{0}$ to determine the number of iterations per curricula. Assume we have *C* curricula and set $max\_iter_{0}=I$. Denote the number of iterations per curriculum by curric_iter. Due to potential uneven division, we cannot simply set $curric\_iter=\frac{max\_iter_{0}}{C}$. For this reason, we set an iteration scheme

$$iter\_vec=\left[\left\lfloor \frac{max\_iter_{0}}{C}\right\rfloor ,\left\lfloor \frac{max\_iter_{0}}{C}\right\rfloor ,\cdots ,\left\lfloor \frac{max\_iter_{0}}{C}\right\rfloor \right]$$

where iter_vec has length *C*. To ensure that the sum of iter_vec is equal to $max\_iter_{0}$, we compute $m=\;\mathrm{mod}\;(max\_iter_{0},C)$ and randomly select *m* elements of iter_vec. We then add one iteration to each selected element. The random selection ensures that there is no bias introduced by this uneven division and the addition of one to *m* elements of iter_vec ensures that the elements of iter_vec sum to *C*.

## Appendix C

Figure A1 shows the 3D structures from CBCR trained on the primary and secondary GM12878 mappings separately for chromosomes 2, 14, and 21 at 1 mb resolution. Figure A2 shows the 3D structures from CBCR trained on the Mbol and DpnII restriction enzyme Hi-C data from GM12878 for chromosomes 2, 14, and 21 at 1 mb resolution. From these figures, one can see that the output for CBCR is qualitatively similar across all chromosomes regardless of the mapping or restriction enzyme.

Figure A3 and Figure A4 show the comparison between the distances associated with looped and non-looped regions in individual chromosomes from the outputs of CBCR from GM12878 at 100kb resolution for the ChIA-PET and HiChIP data respectively.
